# Design of an L-Valine-Modified Nanomicelle-Based Drug Delivery System for Overcoming Ocular Surface Barriers

**DOI:** 10.3390/pharmaceutics14061277

**Published:** 2022-06-16

**Authors:** Huimin Wu, Yuchen Xu, Mengru Cai, Longtai You, Jing Liu, Xiaoxv Dong, Xingbin Yin, Jian Ni, Changhai Qu

**Affiliations:** School of Chinese Material Medica, Beijing University of Chinese Medicine, Beijing 102488, China; 20190935128@bucm.edu.cn (H.W.); 20200935155@bucm.edu.cn (Y.X.); 20210941442@bucm.edu.cn (M.C.); 20190941307@bucm.edu.cn (L.Y.); 20210941441@bucm.edu.cn (J.L.); 201801020@bucm.edu.cn (X.D.); yxbtcm@bucm.edu.cn (X.Y.)

**Keywords:** L-valine, nanomicelles, baicalin, PepT1, endocytosis proteins, tight junction proteins, ocular drug delivery system

## Abstract

The incidence of ocular surface disease (OSD) is increasing, with a trend towards younger ages. However, it is difficult for drugs to reach the deep layers of the cornea due to ocular surface barriers, and bioavailability is less than 5%. In this study, DSPE-PEG2000 was modified with L-valine (L-Val), and an HS15/DSPE-PEG2000-L-Val nanomicelle delivery system containing baicalin (BC) (BC@HS15/DSPE-PEG2000-L-Val) was constructed using thin-film hydration, with a high encapsulation rate, small particle size and no irritation to the ocular surface. Retention experiments on the ocular surface of rabbits and an in vivo corneal permeation test showed that, compared with the control, nanomicelles not only prolonged retention time but also enhanced the ability to deliver drugs to the deep layers of the cornea. The results of a protein inhibition and protein expression assay showed that nanomicelles could increase uptake in human corneal epithelial cells (HCEC) through energy-dependent endocytosis mediated by clathrin, caveolin and the carrier pathway mediated by PepT1 by inhibiting the overexpression of claudin-1 and ZO-1 and suppressing the expression of PepT1-induced by drug stimulation. These results indicate that BC@HS15/DSPE-PEG2000-L-Val is suitable for drug delivery to the deep layers of the ocular surface, providing a potential approach for the development of ocular drug delivery systems.

## 1. Introduction

Due to poor personal habits relating to the use of the eyes, as well as environmental stimulation, the incidence of ocular surface disease (OSD) has been increasing, with a trend towards a younger age group [1,2]. In particular, dry eye disease (DED) has become the most frequent condition observed in ophthalmic clinical practice [3]. DED occurs on the ocular surface. In the course of disease development, T cells migrate to the ocular surface, secreting inflammation markers, particularly IFN-γ and IL-17. These cytokines promote the release of various proinflammatory mediators (including cytokines, chemokines and matrix metalloproteinases) from the corneal epithelium, increasing the access of inflammatory mediators into the stroma, resulting in pain and more severe injury [4]. Baicalin (BC) has been shown to significantly inhibit T cell differentiation and to downregulate the expression of a host of proinflammatory mediators and cytokines, showing clear anti-inflammatory activity [5,6]. Therefore, the effective delivery of drugs to the deep layers of the cornea is key to the anti-inflammatory effect.

Eye drop administration is characterized by high patient compliance, convenience and few side effects, and eye drops account for 90% of ophthalmic preparations [7]. However, there are multiple barriers to the use of the ocular surface for effective drug delivery, including short retention time due to tears [8] and difficult drug delivery into the eye because of tight cell junctions and active transport of drugs into the corneal epithelium [9,10], resulting in less than 5% bioavailability [11]. Ideal ocular drug delivery systems (ODDS) should be able to (1) increase drug retention time on the ocular surface; (2) reduce degradation caused by enzymes in tears; (3) promote permeation through the cornea; and (4) deliver drugs to specific tissues and minimize nonproductive absorption to increase bioavailability [12]. Nanomicelles are considered one of the most promising strategies for topically applied ophthalmic formulations and can increase the aqueous solubility of hydrophobic agents, prolong retention time on the ocular surface and demonstrate better permeation and absorption into tissue. They can also protect the drug from degradation and allow for sustained release at the site of action, guaranteeing a constant therapeutic concentration in the tissue [13].

Kolliphor^®^ HS15, a nonionic surfactant consisting of 70% polyglycol mono- and di-esters of 12-hydroxystearic acid and 30% free polyethylene glycol [14], has been used to develop ODDS due to its excellent corneal penetration properties and ocular surface retention [15]. DSPE-MPEG2000, which is widely used in nanomicelles, contains hydrophilic PEG and hydrophobic DSPE, with a low critical micelle concentration (CMC), good drug-loading stability and a variety of active groups for targeted modification [16,17,18]. Carriers play an important role in overcoming ocular surface barriers and intraocular pharmacokinetics. Among them, PepT1, a peptide carrier, is important for drug absorption, distribution and clearance in the cornea [19] and can transport dipeptides, tripeptides and α-amino acid esters [20,21]. L-valine (L-Val) is an α-amino acid, and with its dipeptide and ester, it is often used for PepT1 targeting modification in prodrugs or carriers [22,23,24,25].

In order to deliver drugs to the deep layers of the cornea to improve their therapeutic effect, we synthesized DSPE-PEG2000-L-Val using an esterification reaction and constructed HS15/DSPE-PEG2000-L-Val nanomicelles containing BC. The results demonstrated that with this novel delivery mechanism, the nanomicelles increased drug uptake in the corneal epithelium through the comprehensive interaction of endocytosis proteins, tight junction proteins and PepT1. In addition, through ocular surface retention and irritation evaluation, we proved the feasibility of BC@HS15/DSPE-PEG2000-L-Val as an ODDS, providing a basis for future research into its efficacy.

## 2. Materials and Methods

### 2.1. Cell Culture and Animals

Human corneal epithelial cells (HCECs) were obtained from FDCC (FDCC-HYN1135, Fudan IBS Cell Center, Shanghai, China) and grown in Dulbecco’s Modified Eagle Medium (DMEM) containing 10% fetal bovine serum (FBS) and 1% penicillin-streptomycin (all from Corning Incorporated, Corning, NY, USA) at 37 °C with 5% CO_2_. The cells were passaged once every two days.

New Zealand rabbits (2.0–2.2 kg) were obtained from Beijing Jinmuyang (Beijing, China). All the animal studies were performed in accordance with the ARVO Statement for the Use of Animals in Ophthalmic and Vision Research and approved by the Institutional Animal Care and Use Committee of Beijing University of Chinese Medicine.

### 2.2. Synthesis of DSPE-PEG2000-L-Val

A total of 1 g of DSPE-PEG2000-NH_2_ (AVT, Shanghai, China) was dissolved into 10 mL of trichloromethane to obtain Solution I. A total of 125.7 mg of Fmoc-L-valine (Shanghai Yuanye Bio-Technology Co., Ltd., Shanghai, China) was dissolved into 2 mL of trichloromethane, with N-(3-dimethylaminopropyl)-N’-ethylcarbodiimide (EDC) and 4-dimethylaminopyridine (DMAP) added and stirred at 0 °C for 1 h to obtain Solution II. Solution II was slowly dripped into Solution I and reacted at 40 °C for 5 h. The crude product DSPE-PEG2000-L-valine-Fmoc was obtained after removing trichloromethane by rotary evaporation under a vacuum. The crude product was dissolved in 5 mL of dichloromethane, with 1 mL of piperidine added to react at room temperature for 30 min; then it was washed twice with water and dried with anhydrous sodium sulfate. After filtration, vacuum distillation and ice ether precipitation, DSPE-PEG2000-L-Val was obtained by vacuum drying at 35 °C.

### 2.3. Characterizations of DSPE-PEG2000-L-Val

Infrared spectroscopy (IR) and ^1^H-nuclear magnetic resonance (^1^H-NMR) were used to analyze the molecular structure to determine whether the products had been successfully synthesized [26]. The structure of DSPE-PEG2000-L-Val was determined by infrared (IR) using a Fourier-transform infrared spectrometer (FTIR, Nicolet iS5, Thermo Fisher, Waltham, MA, USA) and by ^1^H-NMR using a nuclear magnetic resonance spectrometer (ARX-300, Varian, Palo Alto, CA, USA) in CDCl_3_.

### 2.4. Preparation of BC@HS15/DSPE-PEG2000-L-Val Nanomicelles

A mixture of 6 mg of BC (Aladdin, Shanghai, China), 175 mg of HS15 (BASF, Ludwigshafen, Germany) and 75 mg of DSPE-PEG2000-L-Val was dissolved in methanol and evaporated under vacuum conditions at 37 °C for 10 min. The film was hydrated with 5.4 mL phosphate buffer solution (PBS, pH 5.0) and stirred at 37 °C for 2 h. The mixture was centrifuged (Sorvall ST 8R centrifuge, Thermo Fisher, Waltham, MA, USA) at 12,000 rpm at 4 °C for 10 min and filtered through a 0.22 µm membrane to remove undissolved drug.

### 2.5. Characterization of Nanomicelles

#### 2.5.1. Critical Micelle Concentration (CMC)

CMC generally reflects the stability of micelles after solution dilution. HS15 (35 mg) and DSPE-PEG2000-L-Val (15 mg) were dissolved in 25 mL of water and diluted to yield concentrations in the range of 0.06–2000 µg/mL. Each polymer solution was transferred to a vial containing pyrene (Macklin, Shanghai, China). The emission spectra were determined by a fluorescence spectrophotometer (F-4500, Hitachi, Tokyo, Japan) at 335 nm excitation wavelength. The scanning range was 350–500 nm, the scanning speed was 240 nm/min, the excitation and emission slits were 5 nm and 2.5 nm, respectively, the voltage of the photomultiplier tube was 700 V, and the response time was 5 s. The intensities of I_373nm_ and I_384nm_ vibronic bands were evaluated, and the ratios of these intensities were plotted against the logarithm of the concentration of each sample. The CMC was taken as the intersection of two regression lines calculated from the linear portions of the graphs [27].

#### 2.5.2. Drug Loading (DL) and Encapsulation Efficiency (EE)

BC@HS15/DSPE-PEG2000-L-Val (0.1 mL) was dissolved in 10 mL of methanol and placed in an ultrasonic bath for 10 min to completely break up the micelles. The solution was filtered through a 0.22 µm membrane and subjected to high-performance liquid chromatography (HPLC, 1260, Agilent, Santa Clara, CA, USA) using an Agilent ZORBAX SB-C18 column (4.6 × 250 mm, 5 µm). The mobile phase consisted of a mixture of acetonitrile and 0.1% phosphoric acid solution (30:70, *v/v*). The flow rate was 1.0 mL/min, and the detection wavelength was 280 nm. The DL and EE were calculated as shown below in Equations (1) and (2):

(1) $$\mathrm{DL}\;\left(\%\right)=\frac{\mathrm{weight}\;\mathrm{of}\;\mathrm{drug}\;\mathrm{in}\;\mathrm{micelles}}{\mathrm{weight}\;\mathrm{of}\;\mathrm{feeding}\;\mathrm{carriers}\;\mathrm{and}\;\mathrm{drug}}\times 100\%$$

(2) $$\mathrm{EE}\;\left(\%\right)=\frac{\mathrm{weight}\;\mathrm{of}\;\mathrm{drug}\;\mathrm{in}\;\mathrm{micelles}}{\mathrm{weight}\;\mathrm{of}\;\mathrm{feeding}\;\mathrm{drug}}\times 100\%$$

#### 2.5.3. Morphology, Particle Size and Zeta Potential

To evaluate the physical characteristics of nanomicelles, the morphology of BC@HS15/DSPE-PEG2000-L-Val was determined using a transmission electron microscope (TEM, JEM-1400Flash, JEOL, Tokyo, Japan) after five times dilution with water. The size distribution and zeta potential were evaluated with Zetasizer Nano-ZS (Malvern Company, Malvern, UK).

#### 2.5.4. Infrared Spectroscopy (IR), Thermogravimetric Analysis (TGA) and X-ray Diffraction (XRD)

IR, TGA and XRD are used to characterize the encapsulation of drugs in carriers. When the IR characteristic peak of a drug moves, weakens or disappears, the TGA melting point peak disappears and the XRD diffraction peak is covered, the drug is considered to not exist in the form of crystals but to be encapsulated in nanomicelles or in an amorphous form [28,29]. The structures were evaluated using an FTIR. TGA was carried out with a thermogravimetric analyzer (Mettler Toledo, Zurich, Switzerland) under a nitrogen flow from 40 °C to 500 °C. XRD was recorded on an X-ray diffractometer (Rigaku Ultima IV, Rigaku, Tokyo, Japan) over the 2θ range from 5° to 60° at 40 kV and 40 mA.

### 2.6. Formulation Stability

The stability of nanomicelles is critical to protecting the entrapped hydrophobic component in the core [28]. BC@HS15/DSPE-PEG2000-L-Val was made and stored in a liquid state at 4 °C. Its appearance was observed on days 0, 1, 2, 3, 5, 10, 15 and 30. At each time point, the BC concentration in the solution and particle size of the nanomicelles were determined.

### 2.7. In Vitro Drug Release Study

The release behavior of BC@HS15/DSPE-PEG2000-L-Val nanomicelles in PBS (pH 6.0) was investigated using a dynamic dialysis method at 34 °C. Drug-loaded micellar solution and BC solution (containing 1 mg of BC) were transferred to dialysis bags (8–14 KDa). An amount of 1 mL of sample solution was withdrawn at 0.25, 0.5, 0.75, 1, 1.5, 2, 3, 4, 6, 8, 10 and 12 h, and an equal volume of fresh PBS was added. The release concentrations were determined by HPLC, as described in Section 2.5.2. The cumulative release percentage of BC was calculated, and the data were plotted in various kinetic models.

### 2.8. Irritation Studies on Rabbit Ocular Surface

Four rabbits were assessed with a slit lamp microscope (BQ900, Haag Streit, Bern, Switzerland) and determined to have no eye disease present. An amount of 40 µL of BC@HS15/DSPE-PEG2000-L-Val was administered to the conjunctival sac of the left eyes, and 40 µL of physiological saline was administered to the conjunctival sac of the right eyes as a control. The medication was delivered four times per day, with 4 h between treatments for 7 days. A Draize test was carried out with a slit lamp microscope to observe the rabbit’s ocular surface each day and at 1, 2, 4, 24, 48 and 72 h after the last treatment administration to record the ocular conditions, as shown in Tables S1 and S2 [30]. Corneal staining with fluorescein sodium (FLS) was observed under the cobalt blue light of a slit lamp microscope after each Draize test. The cornea, conjunctiva and iris were observed after hematoxylin–eosin (HE) staining and scanned with a Panoramic Desk (3DHistech Panoramic digital slide scanner, 3DHistech, Budapest, Hungary).

### 2.9. Retention Study on Rabbit Ocular Surface

The FLS was dissolved in a micellar solution and PBS (pH 5.0) and then infused into the conjunctival sac of two eyes. Each group was photographed after 1, 3, 5, 10, 15 and 20 min under the cobalt blue light of a slit lamp microscope.

### 2.10. Permeation Study in Rabbit Cornea

HS15/DSPE-PEG2000-L-Val nanomicelles were labeled with coumarin 6 (C_6_, Shanghai Yuanye Bio-Technology Co., Ltd., Shanghai, China) to observe drug distribution behavior, and C_6_ was loaded into the nanomicelles using the same procedure as for BC@HS15/DSPE-PEG2000-L-Val. An amount of 40 µL of C_6_@HS15/DSPE-PEG2000-L-Val was administered in the conjunctival sac of the left eyes and 40 µL of free C_6_ in the conjunctival sac of the right eyes as a control. At fixed time intervals (0, 10, 40 and 90 min), the rabbits were euthanized via air embolism, and their eyes were immediately removed. The corneas were isolated and washed in physiological saline and then sliced vertically along the sagittal plane using a cryostat microtome (NX50, Thermo Fisher, Waltham, MA, USA). The corneal slices were observed and recorded via a laser scanning confocal microscope (Eclipse Ti, Nikon, Tokyo, Japan).

### 2.11. In Vitro Cytotoxicity Assay

A CCK-8 assay was used to evaluate the toxicity of BC and BC@HS15/DSPE-PEG2000-L-Val on HCECs. The cells were inoculated into 96-well plates (1 × 10^4^ cells/well) and cultured for 24 h at 37 °C. BC with different concentrations (1, 2, 4, 6 and 8 µg/mL), and nanomicelles with different diluted multiples (500, 400, 300, 200 and 100) were added. After 2 h, CCK-8 was treated for another 1 h. Absorbance was measured at 450 nm with a microplate analyzer (Epoch2, BioTek, VT, USA).

### 2.12. Cellular Uptake Studies

HCECs were inoculated into 6-well plates (2.5 × 10^5^ cells/well) and cultured for 24 h. The cells were treated with C_6_ solution and C_6_@HS15/DSPE-PEG2000-L-Val (0.1 µg/mL C_6_) for 10, 30, 60 and 120 min, followed by washing twice with PBS. The HCECs were then photographed through a fluorescence inverted microscope (Eclipse Ts2R, Nikon, Tokyo, Japan) at 470 nm excitation wavelength. The mean intracellular fluorescence intensity was measured with a flow cytometer (BD FACSCanto II, BD, Franklin Lakes, NJ, USA) at 488 nm excitation wavelength.

### 2.13. Uptake Mechanisms Studies

#### 2.13.1. Endocytosis Proteins

Endocytosis is an important method for cell uptake of nanoparticles [31] and is usually mediated by clathrin and caveolin and is energy dependent. For protein inhibition, HCECs were inoculated into 6-well plates (2.5 × 10^5^ cells/well) and cultured for 24 h. The cells were preincubated with chlorpromazine hydrochloride (6 µg/mL), indomethacin (36 µg/mL) and amilorid HCl dihydrate (3 µg/mL) for 30 min at 37 °C and then treated with C_6_@HS15/DSPE-PEG2000-L-Val (0.1 µg/mL C_6_, containing the same concentration of inhibitors) for 2 h at 37 °C. For energy inhibition, the cells were preincubated at 4 °C and then treated with C_6_@HS15/DSPE-PEG2000-L-Val (0.1 µg/mL C_6_) at 4 °C. The mean intracellular fluorescence intensity was measured with a flow cytometer.

#### 2.13.2. Tight Junction Proteins

Western blotting (WB) was used to detect the effect of nanomicelles on tight junction proteins. HCECs were inoculated into 6-well plates (2.5 × 10^5^ cells/well) and cultured for 24 h. The cells were treated with BC and BC@HS15/DSPE-PEG2000-L-Val (4 µg/mL BC) for 2 h. Then, the treated cells were washed twice with cold PBS and lysed with RIPA buffer for 30 min. The lysate was centrifuged at 4 °C for 5 min (12,000 rpm), heated at 100 °C for 5 min, separated by 10% SDS-PAGE and transferred to a PVDF membrane with a TBST buffer containing 5% skimmed milk. The membrane was sealed for 1 h and incubated overnight at 4 °C with a primary antibody (claudin-1 (1:500), ZO-1 (1:500) or GAPDH (1:1000)). Then, it was incubated with horseradish peroxidase-conjugated secondary antibodies (1:8000) at room temperature for 2 h. An enhanced chemiluminescence (ECL) reagent was used to visualize the bands.

#### 2.13.3. PepT1

Glycylsarcosine (Gly-Sar) was used as a PepT1 inhibitor to confirm the targeting effect of nanomicelles according to the experimental procedure described in Section 2.13.1. WB was used to detect the effect of nanomicelles on PepT1 in accordance with the procedure described in Section 2.13.2.

### 2.14. Statistical Methods

All experiments were performed at least in triplicate, and the data were expressed as mean ± SD. Statistical analysis was performed by one-way ANOVA; * *p* < 0.05, ** *p* < 0.01 and *** *p* < 0.001 were considered to be statistically significant.

## 3. Results

### 3.1. Synthesis and Characterization of DSPE-PEG2000-L-Val

**Figure 1:**
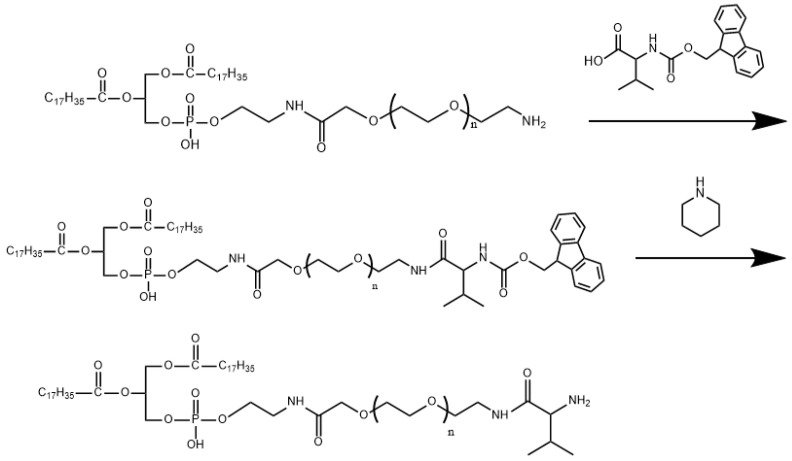
Synthetic scheme of DSPE-PEG2000-L-Val.

**Figure 2:**
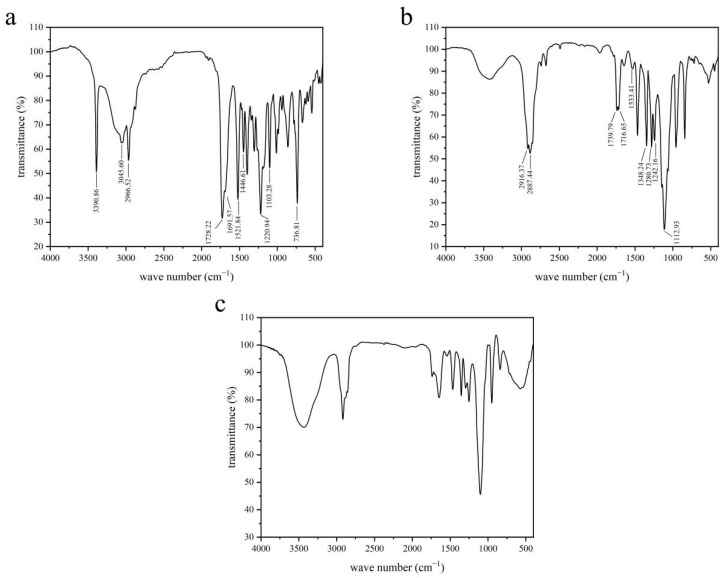
IR spectra of (**a**) Fmoc-L-Val, (**b**) DSPE-PEG2000-NH_2_ and (**c**) DSPE-PEG2000-L-Val.

**Figure 3:**
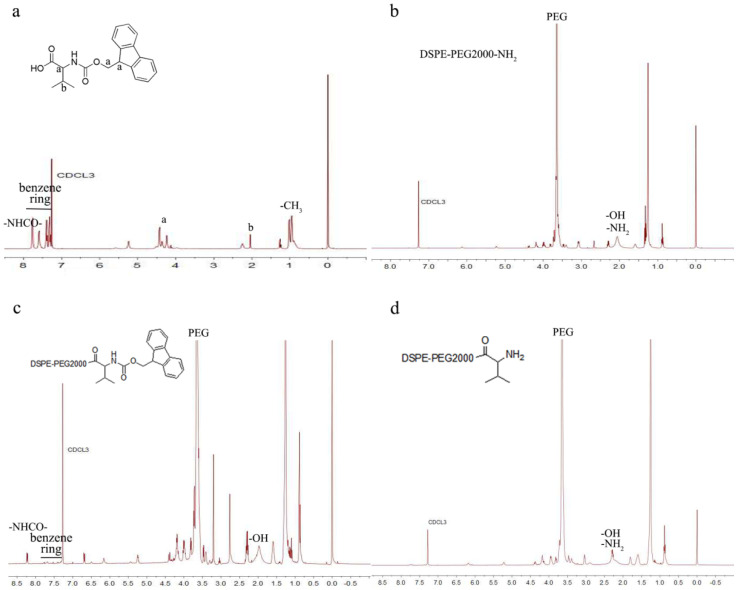
^1^H-NMR spectra of (**a**) Fmoc-L-Val, (**b**) DSPE-PEG2000-NH_2_, (**c**) DSPE-PEG2000-L-Val-Fmoc and (**d**) DSPE-PEG2000-L-Val.

The synthesis of DSPE-PEG2000-L-Val is presented in Figure 1. The IR spectra of Fmoc-L-Val, DSPE-PEG2000-NH_2_ and DSPE-PEG2000-L-Val are presented in Figure 2. Overall, the spectrum of DSPE-PEG2000-L-Val was similar to that of DSPE-PEG2000-NH_2_; however, the wide absorption peak at 3600–3000 cm^−1^ was obviously enhanced, which may have been influenced by the N-H stretching vibration introduced by L-Val. The ^1^H-NMR spectra of Fmoc-L-Val, DSPE-PEG2000-NH_2_, DSPE-PEG2000-L-Val-Fmoc and DSPE-PEG2000-L-Val are presented in Figure 3. DSPE-PEG2000-L-Val-Fmoc had a characteristic proton peak of PEG at 3.6 ppm from DSPE-PEG2000-NH_2_ [18], a proton peak of -NHCO- at 8.2 ppm and proton peaks of benzene rings at 7.0–8.0 ppm from Fmoc, indicating that Fmoc-L-Val and DSPE-PEG2000-NH_2_ were linked. The signals attributed to Fmoc disappeared in DSPE-PEG2000-L-Val, indicating that Fmoc was removed. The target product was successfully synthesized.

### 3.2. Characterization and Stability of BC@HS15/DSPE-PEG2000-L-Val

**Figure 4:**
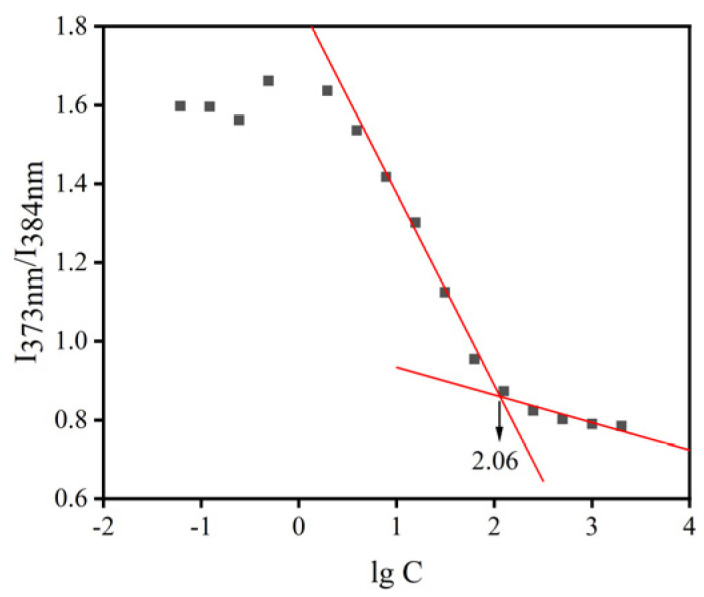
CMC of mixed micellar materials. (The red lines represents regression lines).

The normal volume of tears in the conjunctival sac of humans is about 7 µL, and the average volume of eye drops is 40 µL [27]. The intensity ratio of HS15/DSPE-PEG2000-L-Val is shown in Figure 4, and the CMC value was 114.82 µg/mL, indicating that the nanomicelles had good stability in the aqueous media.

**Figure 5:**
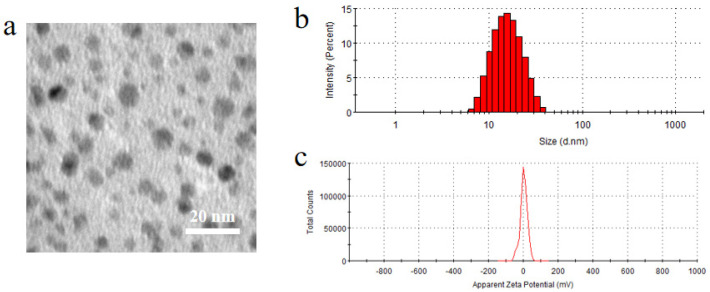
(**a**) Morphology, (**b**) particle size and (**c**) zeta potential of BC@HS15/DSPE-PEG2000-L-Val.

As shown in Figure 5, the nanomicelles exhibited almost spherical and uniform shape with dark solid spheres and were distributed homogeneously. The mean particle size was 19.45 ± 0.50 nm with a zeta potential value of 1.44 ± 1.61 mV, and the drug loading and encapsulation efficiency of nanomicelles were 2.23 ± 0.04% and 99.97 ± 2.19%, respectively.

**Figure 6:**
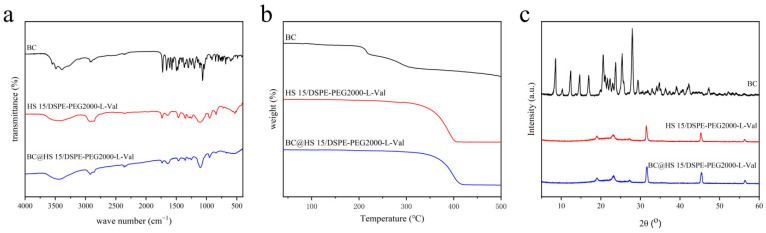
(**a**) IR spectra, (**b**) TGA patterns and (**c**) XRD patterns of BC, HS15/DSPE-PEG2000-L-Val and BC@HS15/DSPE-PEG2000-L-Val.

The IR spectrum of drug-loaded nanomicelles was largely consistent with that of blank nanomicelles, while some characteristic peaks of BC were covered (Figure 6a). As shown in the TGA pattern (Figure 6b), the weight of BC began to change significantly at about 200 °C, while that of nanomicelles began to change at about 350 °C. As shown in the XRD pattern (Figure 6c), the typical diffraction peaks of BC were obvious in the range of 5–30°, which disappeared in the nanomicelles. These data demonstrate that the crystallinity of BC was good, while it was in an amorphous state in BC@HS15/DSPE-PEG2000-L-Val, indicating that the drug was encapsulated in nanomicelles.

**Figure 7:**
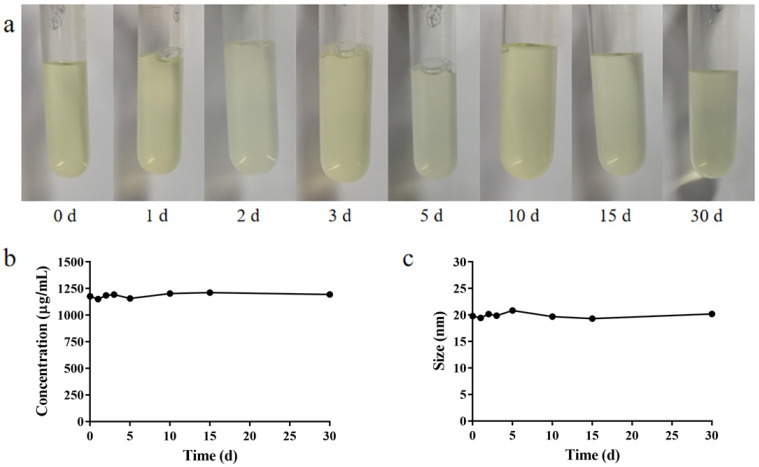
(**a**) Appearance, (**b**) BC concentration in the solution and (**c**) particle size of BC@HS15/DSPE-PEG2000-L-Val.

The nanomicelle solution was light yellow and transparent, and there were no obvious changes in appearance, drug concentration or particle size when stored at 4 °C for 30 days (Figure 7), indicating that the nanomicelles could be stored at 4 °C for at least 30 days without deterioration.

### 3.3. In Vitro Drug Release Study

**Figure 8:**
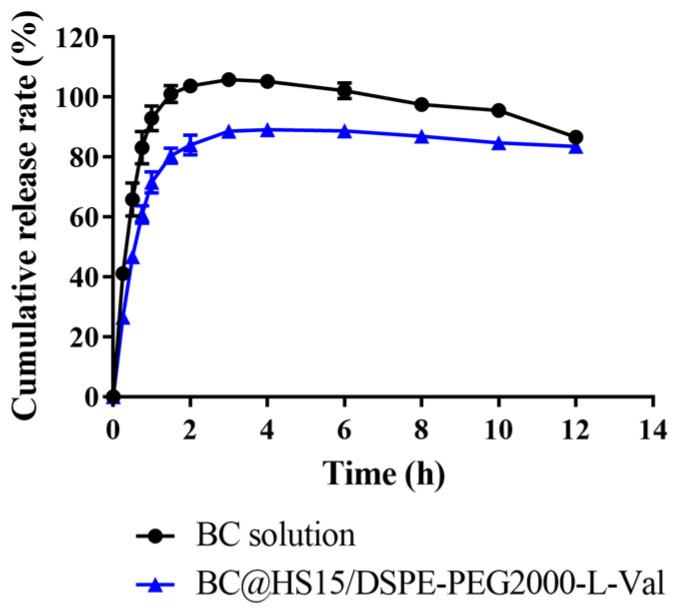
The cumulative release of BC from solution and nanomicelles in PBS at pH 6.0 (*n* = 3).

The in vitro release of BC from different formulations was investigated. As shown in Figure 8, the cumulative release of BC from solution showed a decreasing trend after reaching the maximum at 3 h, while that of BC from nanomicelles remained unchanged from 4 h to 12 h, indicating that BC was unstable in PBS (pH 6.0), while it was continuously released from nanomicelles to maintain drug concentration. The data in Table 1 also demonstrated that the solution and nanomicelles had similar release trends and were most consistent with the first order model. According to the Ritger–Peppas model, the release behavior of BC in solution was in accordance with Fick diffusion (K < 0.45), while in nanomicelles, it was a combination of diffusion and skeleton dissolution (0.45 < K < 0.89), which explained why BC released more slowly in the nanomicelles.

### 3.4. Irritation Studies on Rabbit Ocular Surface

**Figure 9:**
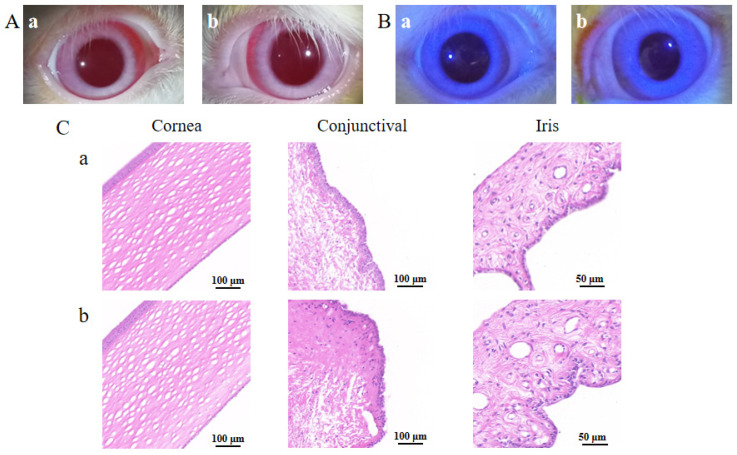
The irritation results of (**A**) Draize test, (**B**) FLS staining test on 7th day, and (**C**) histopathological sections of cornea, conjunctiva and iris in (**a**) physiological saline and (**b**) BC@HS15/DSPE-PEG2000-L-Val.

A representative result of the Draize test is shown in Figure 9A. The cornea, conjunctiva and iris were clear, with no congestion or edema, and an occasional small amount of secretion was observed in the corner of the eyes in both the physiological saline group and the BC@HS15/DSPE-PEG2000-L-Val group. The eye irritation response score at various time points was ≤1, indicating no irritation. The representative result of the FLS staining test is shown in Figure 9B. There were less than five punctate stains observed in the cornea in the two groups, indicating that the nanomicelles caused no damage to the cornea after multiple administrations. The representative result of HE staining is shown in Figure 9C. Compared with the physiological saline group, the cornea, conjunctiva and iris were intact, with clear boundaries between the layers, and there was no obvious inflammatory-cell infiltration or hyperplasia and no edema or histopathological changes in the three tissues. Thus, BC@HS15/DSPE-PEG2000-L-Val did not produce eye irritation, making it suitable for ocular applications.

### 3.5. Retention Study on Rabbit Ocular Surface

**Figure 10:**
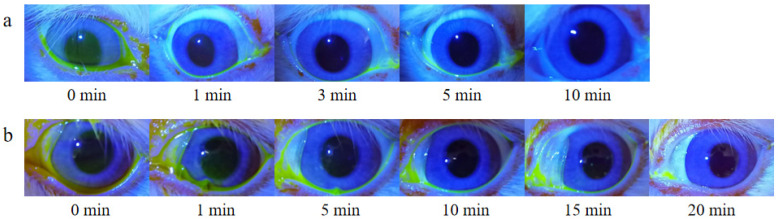
In vivo fluorescence imaging of rabbit ocular surfaces at various time points post-dropping of (**a**) FLS solution and (**b**) FLS nanomicelles.

Due to the blink reflex and tear dilution, FLS gradually decreased until it disappeared completely through nasolacrimal duct drainage. As shown in Figure 10, the disappearance time of the fluorescent layer in the FLS solution group was about 5–10 min, and in the FLS nanomicelles this was extended to 15–20 min, indicating that the nanomicelles could prolong the retention time on the ocular surface effectively.

### 3.6. Permeation Study in Rabbit Cornea

**Figure 11:**
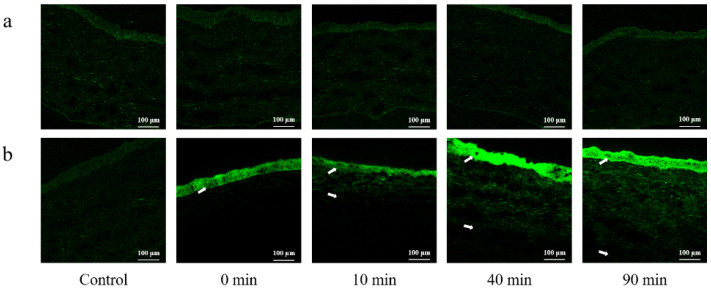
Confocal laser scanning microscopy of rabbit corneal tissues at various time points post-dropping of (**a**) free C_6_ and (**b**) C_6_@HS15/DSPE-PEG2000-L-Val. (The white arrow represents where C_6_ penetrated into the cornea).

Confocal laser scanning microscopy was carried out to observe the capacity of different preparations labeled by C_6_ on corneal permeation. As shown in Figure 11, there was no obvious green fluorescence observed in the cornea at any time point after the free C_6_ was administered, indicating that it was difficult for C_6_ to penetrate the cornea. However, bright green fluorescence was observed in the corneal epithelial layer at 0 min after C_6_@HS15/DSPE-PEG2000-L-Val was administered. As time progressed, the corneal stroma layer also appeared fluorescent, and the penetration depth gradually increased. The results demonstrated that C_6_ could rapidly penetrate the corneal epithelium and gradual penetrate into the stroma with the help of nanomicelles, sustaining the hypothesis that nanomicelles can effectively enhance the permeability of BC into the cornea.

### 3.7. In Vitro Cytotoxicity Assay

**Figure 12:**
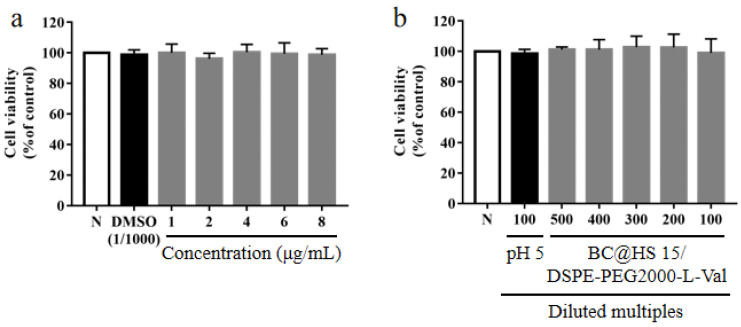
The survival rate of HCECs after 2 h incubation with different concentrations of (**a**) BC and (**b**) different diluted multiples of BC@HS15/DSPE-PEG2000-L-Val (N; the control group, *n* = 3).

To evaluate the safety of BC@HS15/DSPE-PEG2000-L-Val, a CCK-8 experiment was employed. The results revealed that compared with the control group, there was no significant difference in the survival rate of the HCECs after 2 h post administration of BC (1–8 µg/mL) and BC@HS15/DSPE-PEG2000-L-Val (100–500 times dilution) (*p* > 0.05, Figure 12), indicating that neither BC nor the nanomicelles in this concentration range had cytotoxicity to the HCECs within 2 h.

### 3.8. Cellular Uptake Studies

**Figure 13:**
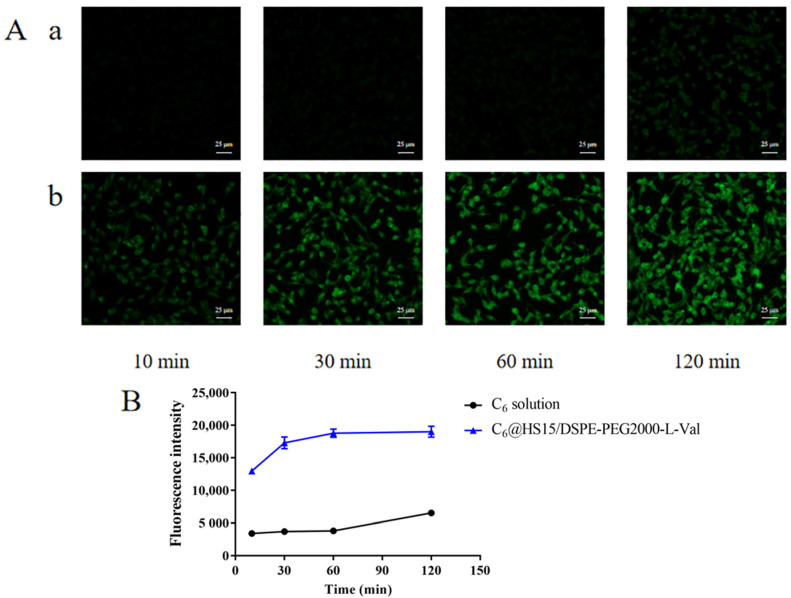
Cellular uptake of C_6_ by HCECs: (**A**) fluorescence inverted microscope observations of cell uptake characteristics and (**B**) flow cytometry determination of cell uptake characteristics; (**a**) C_6_ solution and (**b**) C_6_@HS15/DSPE-PEG2000-L-Val (*n* = 3).

Fluorescence inverted microscope observations showed very little green fluorescence on the HCECs within 60 min after C_6_ solution administration. Weak fluorescence appeared at 120 min, and the fluorescence could be observed 10 min after C_6_@HS15/DSPE-PEG2000-L-Val administration. Intensity increased significantly with extension of incubation time, presenting an obvious time-dependence (Figure 13A). Flow cytometry determination results showed that cellular uptake of the C_6_ solution was low within 60 min and increased at 120 min but was still at a low level. In the C_6_@HS15/DSPE-PEG2000-L-Val group, the fluorescence intensity increased significantly at each time point in a time-dependent manner and reached the complete uptake state at 60 min (Figure 13B). These results showed that the HCECs had difficulty taking up free C_6_, but it could be enhanced in C_6_@HS15/DSPE-PEG2000-L-Val, indicating that the nanomicelles were rapidly and time-dependently taken up by the HCECs.

### 3.9. Uptake Mechanisms Studies

**Figure 14:**
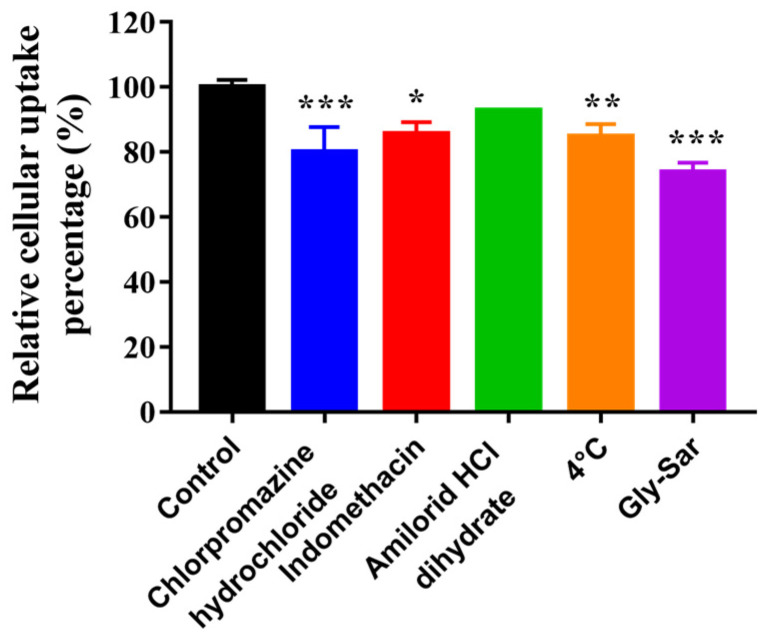
Effects of different inhibition conditions on cellular uptake of C_6_ by HCECs (*n* = 3; * *p* < 0.05, ** *p* < 0.01 and *** *p* < 0.001 significantly different compared with control).

Flow cytometry was carried out to determine the effects of different inhibition conditions on the cellular uptake of C_6_ by HCECs. As shown in Figure 14, the relative cellular uptake percentage of the control group was 100.00 ± 1.86%, chlorpromazine hydrochloride (clathrin inhibitor) was 80.08 ± 6.30%, indomethacin (caveolin inhibitor) was 85.60 ± 2.94%, amilorid HCl dihydrate (macropinocytosis inhibitor) was 92.76 ± 0.25%, at 4 °C (energy inhibition), it was 84.85 ± 3.09% and with Gly-Sar (PepT1 inhibitor), it was 73.83 ± 2.36%. Chlorpromazine hydrochloride, indomethacin, 4 °C condition and Gly-Sar significantly inhibited the uptake of the nanomicelles (*p* < 0.05), indicating that the cellular uptake of the drug in the nanomicelles was related to clathrin- and caveolin-mediated, energy-dependent endocytosis and PepT1-mediated active transport.

**Figure 15:**
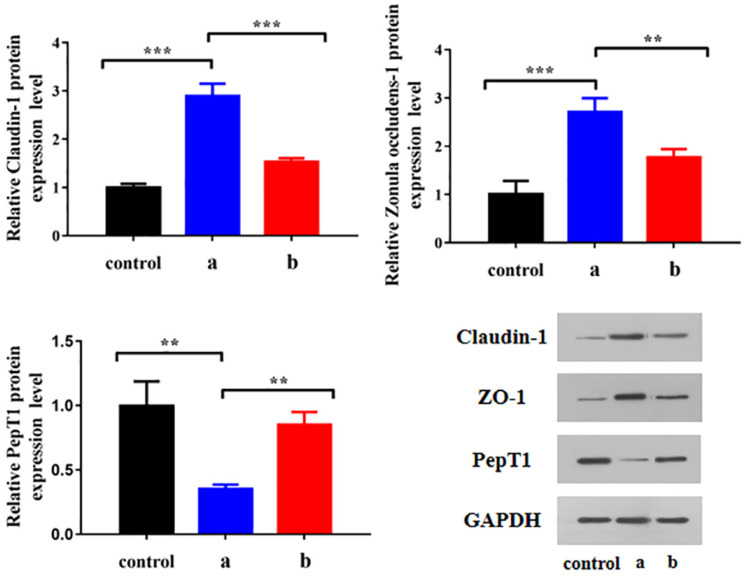
Changes of claudin-1, ZO-1 and PepT1 protein expression level in HCECs cultured with (a) BC solution and (b) BC@HS15/DSPE-PEG2000-L-Val (*n* = 3; ** *p* < 0.01, *** *p* < 0.001).

WB was carried out to determine the changes to claudin-1, ZO-1 and PepT1 protein expression levels in HCECs cultured with different preparations. As shown in Figure 15, after 4 µg/mL BC administration, the expression levels of claudin-1 and ZO-1 in HCECs were significantly increased and that of PepT1 was significantly decreased (*p* < 0.01), while these trends were inhibited after BC@HS15/DSPE-PEG2000-L-Val intervention (*p* < 0.01), indicating that BC upregulated the expression of tight junction proteins and downregulated PepT1 in HCECs, making the penetration and cellular uptake of drugs more difficult, while nanomicelles alleviated the adverse effects of BC, which promoted drug delivery to the deep layers of the cornea.

## 4. Discussion

The antioxidant and anti-inflammatory effects of BC, a flavonoid, have been demonstrated in various disease models, including neurodegenerative, liver and kidney diseases, diabetes, cardiovascular diseases, rheumatoid arthritis and asthma [32]. In addition, BC has benefits in the treatment of ocular diseases, such as ocular inflammation, cataracts, glaucoma and diabetic retinopathy [5,6]. However, BC is a BCS class IV compound with poor solubility and permeability (P_app_ = 0.37 × 10^−6^ cm/s), which directly affects the dissolution rate and transmembrane transportation rate [33], resulting in low bioavailability [34]. Furthermore, BC is sensitive to pH [35]. An in vitro drug release study demonstrated that the concentration of BC in PBS (pH 6.0) decreased gradually. In our study, the solubility and stability of BC was improved through nanomicelle encapsulation.

Size and surface charge of nanomicelles are important factors in the design of ODDS. Particle size affects cellular uptake processes, such as endocytosis, which may be the main mechanism of drug uptake by corneal and conjunctiva cells. The smaller the size, the higher the permeability [36,37]. Surface charge affects micelle interaction with cells. Mucins on the ocular surface are negatively charged at the physiological pH and, therefore, have a stronger affinity for positively charged ligands. Moreover, hydroxyl, carboxyl and sulfhydryl groups on the polysaccharide branch chain of the mucins provide abundant surface area for hydrogen bond formation. Therefore, polar hydrophilic molecules such as polymers can enhance ocular surface adhesion. Moreover, adhesion can also be achieved through physical chain entanglement between polymers and mucin fibers, prolonging ocular surface retention time [38,39]. In this study, HS15 demonstrated good performance for corneal retention [15]. DSPE-PEG2000-L-Val is a polymer material with a positive charge, and the average particle size of BC@HS15/DSPE-PEG2000-L-Val is less than 20 nm, which enhanced the ocular surface adhesion, prolonged the retention time and increased the corneal permeation of drugs.

Endocytosis is mostly demonstrated by the addition of inhibitors. Common clathrin inhibitors include chlorpromazine and sucrose; common caveolin inhibitors include indomethacin, nystatin, methyl-β-cyclodextrin and genistein; common macropinocytosis pathway inhibitors include amiloride, cytochalasin B and colchicine; and common forms of ATP energy suppression include sodium azide, 2-deoxyglucose and low temperature [40,41,42]. In this study, HCECs were co-incubated with chlorpromazine, indomethacin, amiloride, at 4 °C and with nanomicelles, separately, proving that the cellular uptake mechanism was energy-dependent, clathrin- and caveolin-mediated endocytosis.

At present, the main corneal tight junction proteins studied are ZO proteins (ZO-1, ZO-2 and ZO-3), occludins and claudins (claudins 1, 2, 3, 4, 7, 9, 14 and 15). In surface cells, ZO proteins and claudins are mainly expressed. ZO-1 is the marker protein of tight junctions in the corneal epithelium, and claudin-1 is the main protein expressed in claudins [43,44,45]. The effects of ophthalmic preparations on tight junction proteins can be determined by PCR, WB or immunofluorescence staining [45,46,47]. In this study, the expression changes of ZO-1 and claudin-1 were detected with WB, proving that the cellular uptake mechanism of nanomicelles inhibits the overexpression of ZO-1 and claudin-1 induced by drug stimulation, which is a novel and noteworthy discovery.

PepT1 is the targeted receptor of L-Val, which was verified mostly by adding competitive inhibitor Gly-Sar [25,48,49]. In this study, HCECs were co-incubated with Gly-Sar and nanomicelles, and changes to PepT1 expression were detected with WB, proving that the cellular uptake mechanism was PepT1-mediated active transport and that it improved the inhibition of PepT1 induced by drug stimulation.

The study results showed that BC induced the upregulation of tight junction protein expression in corneal epithelial cells. This phenomenon occurred due to the stimulation of the ocular surface by 15 mM glucose, which may be a compensatory cellular response to negative stress [45]. However, few studies have reported stimulation by therapeutic agents, which not only reflected the advantage of using nanomicelles to deliver drugs but also provided a new idea for studying the ocular delivery obstacles of ophthalmic drugs in the future.

## 5. Conclusions

In this research, we constructed a novel ODDS, BC@HS15/DSPE-PEG2000-L-Val nanomicelles, to enhance drug delivery into the deepest layer of cornea. The nanomicelles effectively encapsulated BC and exhibited good drug release performance, prolonged ocular surface retention time and had no stimulation after constantly repeated administration. Furthermore, the constructed nanomicelles increased corneal permeation and cellular uptake of the drug by affecting endocytosis proteins (clathrin and caveolin), tight junction proteins (claudin-1 and ZO-1) and the carrier protein PepT1. In conclusion, an ODDS based on L-Val modified nanomicelles meeting the requirements of ophthalmic preparations was designed and constructed, providing a beneficial exploration for promoting drug enrichment in the corneal deep layers through a multiple interaction approach.

## Figures and Tables

**Table 1:** Mathematical model fitting results.

Model Equations	Solution	Nanomicelles
zero order	M_t_/M_∞_ = 0.3204t + 0.443 (R^2^ = 0.7779)	M_t_/M_∞_ = 0.2262t + 0.4447 (R^2^ = 0.7449)
first order	ln (1 − M_t_/M_∞_) = −2.0149t − 0.0019 (R^2^ = 0.9966)	ln (1 − M_t_/M_∞_) = −1.7096t − 0.1548 (R^2^ = 0.9849)
Higuchi	M_t_/M_∞_ = 0.6643t^1/2^ + 0.131 (R^2^ = 0.8842)	M_t_/M_∞_ = 0.5546t^1/2^ + 0.1458 (R^2^ = 0.8735)
Ritger–Peppas	ln (M_t_/M_∞_) = 0.401lnt − 0.2667 (R^2^ = 0.8552)	ln (M_t_/M_∞_) = 0.4809lnt − 0.3631 (R^2^ = 0.9055)

## Data Availability

The data presented in this study are available on request from the cerresponding authors.

## Supplementary Materials

The following supporting information can be downloaded at: https://www.mdpi.com/article/10.3390/pharmaceutics14061277/s1, Table S1: Eye irritation response score standard; Table S2: Evaluation criteria of eye irritation.
