# Correction: Wu et al. Design of an L-Valine-Modified Nanomicelle-Based Drug Delivery System for Overcoming Ocular Surface Barriers. *Pharmaceutics* 2022, *14*, 1277

**DOI:** 10.3390/pharmaceutics18070810

**Published:** 2026-06-30

**Authors:** Huimin Wu, Yuchen Xu, Mengru Cai, Longtai You, Jing Liu, Xiaoxv Dong, Xingbin Yin, Jian Ni, Changhai Qu

**Affiliations:** School of Chinese Material Medica, Beijing University of Chinese Medicine, Beijing 102488, China; 20190935128@bucm.edu.cn (H.W.); 20200935155@bucm.edu.cn (Y.X.); 20210941442@bucm.edu.cn (M.C.); 20190941307@bucm.edu.cn (L.Y.); 20210941441@bucm.edu.cn (J.L.); 201801020@bucm.edu.cn (X.D.); yxbtcm@bucm.edu.cn (X.Y.)

## Error in Figure

In the original publication [1], there was a mistake in Figure 11 as published. The error occurred during the preparation of Figure 11, where the a-control was mistakenly pasted as a-0 min.

The corrected Figure 11 appears below. The authors state that the scientific conclusions are unaffected. This correction was approved by the Academic Editor. The original publication has also been updated.

## Figures and Tables

**Figure 11:**
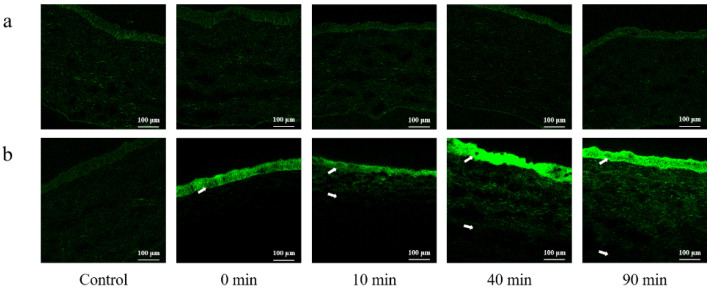
Confocal laser scanning microscopy of rabbit corneal tissues at various time points post-dropping of (**a**) free C_6_ and (**b**) C_6_@HS15/DSPE-PEG2000-L-Val. (The white arrow represents where C_6_ penetrated into the cornea).
