# Supplementary material for: Design of an L-Valine-Modified Nanomicelle-Based Drug Delivery System for Overcoming Ocular Surface Barriers
Source: Pharmaceutics. 2022 Jun 16;14(6):1277. doi: 10.3390/pharmaceutics14061277 (PMC9230556; doi:10.3390/pharmaceutics14061277)
Supplement: Supplementary file 1 [file pharmaceutics-14-01277-s001.zip › pharmaceutics-1739394-supplementary.pdf]

Table S1. Eye irritation response score standard

Factors	Eye stimulation response.	Score
Conjunctival congestion	Clear blood vessels with reddish color	0
	Mildly congested blood vessels with bright red color	1
	Congested blood vessels with fuchsia color, not easy to distinguish	2
	Diffuse hyperemia with fuchsia color, with ciliary hyperemia	3
Conjunctival edema	No edema	0
	Mild edema	1
	Obvious edema, with partial ectropion	2
	Edema to nearly half-closed eyelids	3
	Edema to the extent that the eyelids are half closed	4
Conjunctival secretions	No secretion	0
	Small amount of secretions	1
	The secretions make the eyelids and eyelashes moist or sticky	2
	The secretions make the entire eye area moist or sticky	3
Cornea	Clear and transparent cornea without turbidity	0
	Scattered or diffuse light turbidity, visible iris texture	1
	Easy to distinguish the translucent area, with blurred iris	2
	Translucent cornea, blurred iris, and pupil size is barely visible	3
	Completely cloudy cornea and unrecognizable iris	4
Iris	Normal	0
	Obviously deepened wrinkles, hyperemia, swelling, and mild hyperemia around the cornea; the pupils still respond to light	1
	Bleeding, visible necrosis, no response to light or one of the reactions	2
Highest total score		16

Table S2. Evaluation criteria of eye irritation.

Score	Evaluation
0~3	No irritation
4~8	Mild irritation
9~12	Moderate irritation
13~16	Severe irritation
